# Apoptotic Cells Deliver Processed Antigen to Dendritic Cells for Cross-Presentation

**DOI:** 10.1371/journal.pbio.0030185

**Published:** 2005-04-26

**Authors:** Nathalie E Blachère, Robert B Darnell, Matthew L Albert

**Affiliations:** **1**Laboratoire d'Immunobiologie des Cellules Dendritiques, Institut PasteurParisFrance; **2**The Laboratory of Molecular Neuro-oncology, The Rockefeller UniversityNew York, New YorkUnited States of America; **3**Howard Hughes Medical Institute, The Rockefeller UniversityNew York, New YorkUnited States of America; **4**Institut National de la Santé et de la Recherche Médicale (INSERM) AV0201ParisFrance; University of MinnesotaUnited States of America

## Abstract

Antigen derived from engulfed apoptotic cells can be cross-presented by dendritic cells (DCs) for the generation of major histocompatibility class I/peptide complexes. While the early events of recognition and internalization of the dying cell have been characterized, the antigen-processing pathway or pathways remain unknown. We established a mouse model assaying for the activation of polyclonal T cells reactive to antigen derived from apoptotic cells, and demonstrated two distinct pathways for the trafficking of exogenous epitopes. In the first, exogenous antigen is dependent on the DC's expression of a functional transporter associated with antigen processing (TAP). Surprisingly, we found evidence that a second pathway exists in which transfer of processed antigen from the dying cell allows formation of major histocompatibility class I/peptide complexes in TAP-deficient DCs. In vivo data suggest that in situations of stress (e.g., viral infection), this latter pathway may be more efficient, illustrating that dying cells may preselect immunologically important antigenic determinants.

## Abstract 2

When body cells are killed by pathogens such as viruses, professional antigen-presenting cells can alert the immune system to the presence of the invader through two distinct cross-presentation pathways

## Introduction

Apoptosis is considered the primary means by which physiologic cell death occurs [1]. The fate of apoptotic material is rapid clearance and degradation by phagocytes. There is, however, growing evidence that apoptotic death need not be an endpoint, and that dying cells are capable of transferring antigen to the immune system for the induction of T cell immunity [2,3]. We have previously demonstrated that human dendritic cells (DCs) phagocytose apoptotic cells, and rather than degrading the internalized material, the DCs are capable of generating peptide epitopes for major histocompatibility (MHC) I molecules and activating viral- and tumor-antigen-specific CD8^+^ T cells [4–6]. This pathway has been referred to as cross-presentation for its ability to “cross” classically defined restrictions for MHC I antigen presentation [7]. Our work has offered a physiologically relevant mechanism for the in vivo phenomenon of cross-presentation, which accounts for both the cross-priming and cross-tolerization of tissue-restricted antigen-specific CD8^+^ T cells [8–10]. We have demonstrated that antigen capture occurs via receptor-mediated phagocytosis [5,11], and that internalized apoptotic material can be located within the MHC II−containing compartment [12]; however, the trafficking of antigen from the apoptotic cell to the DC for generation of MHC I/peptide (MHC I/pep) complexes has not been fully characterized.

To define the cellular machinery required for cross-presentation, several studies have focused on the use of cells expressing vector-encoded gene products to test whether proteasomal substrates (e.g., intact proteins) or chaperoned peptides serve as the source of antigen [13–15]. While these studies conclude that cellular proteins are the major source of antigen transferred to the immune system, the inability to demonstrate transfer of cell-associated antigen, or the lack of processed antigen within the dying cell, may have skewed the observed results. In other studies, the use of exogenous antigen bound to latex beads [16,17], derived from internalized immune complexes [18] or whole protein [19], does not permit antigen processing to occur prior to capture by an antigen-presenting cell (APC); therefore, it is not surprising that these models demonstrate that the phagosome-to-cytosol pathway is the dominant means by which antigen is trafficked for processing and presentation onto MHC I.

To examine whether antigen processed within the dying cell can be transferred to the DC, we designed in vitro and in vivo experiments to track the activation of polyclonal influenza-reactive T cell responses stimulated by DCs cross-presenting antigen from haplotype-mismatched apoptotic cells. We report the utilization of two independent pathways by which internalized antigen may access MHC I within the DC: in one, the substrate for cross-presentation is whole or partially degraded protein, which must be further processed by the DC; and in the other, we find evidence for processed antigen accessing the MHC I pathway of the DC. Concerning the in vivo presentation of viral antigen, this latter pathway seems dominant, thus permitting efficient loading of MHC I/pep complexes by the DC.

## Results

In order to dissect the pathway or pathways by which antigens derived from apoptotic cells are processed and presented by DCs, we established in vivo and in vitro systems that permit monitoring of direct presentation and cross-presentation of antigen (Protocol S1; Figure S1). Using these model systems, we tested the hypothesis that dying cells participate in the processing of antigen for cross-presentation. To restrict the DCs' capacity to process antigen, we employed bone-marrow-derived DCs prepared from mice deficient in TAP-1. The generation of antigen-specific MHC I/pep complexes was assayed based on the ability to stimulate influenza-reactive T cells. In all experiments, interleukin-12 (IL-12) was added to the DC/CD8^+^ T cell cultures to bypass the requirement for CD4^+^ T cell help [8].

**Figure 1:**
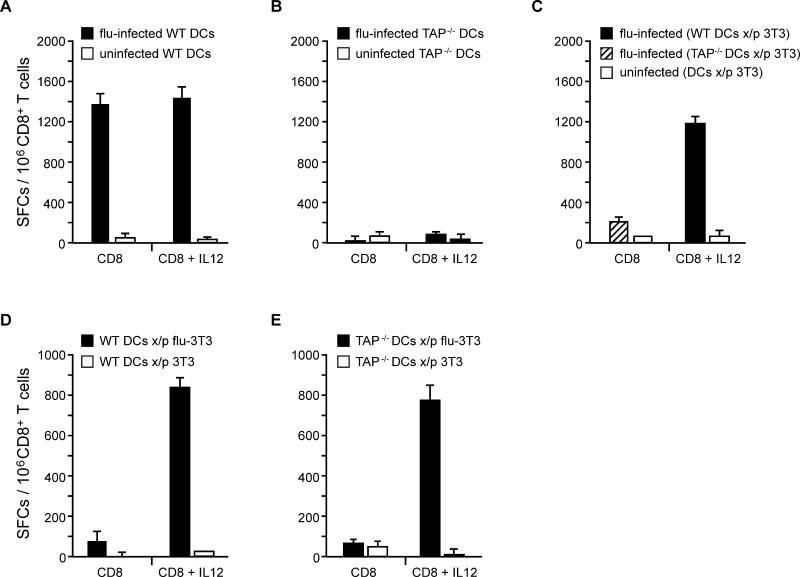
TAP^−/−^ DCs Cross-Present Antigen Derived from Apoptotic Cells (A and B) Antigen presentation via the endogenous pathway was evaluated in WT DCs and TAP^−/−^ DCs by directly infecting cells with influenza virus and assaying for T cell activation. IFN-γ production and T cell precursor frequency were determined using an ELISPOT assay. (C) To evaluate transfer of TAP activity from the dying cells to DCs, WT or TAP^−/−^ DCs after capture of apoptotic cells were directly infected and tested for their respective ability to activate CD8^+^ T cells via the endogenous pathway. (D and E) WT DCs (D) or TAP^−/−^ DCs (E) were co-cultured for 36–48 h with influenza-infected or uninfected allogeneic cells in the presence of TNF-α. As above, mature DCs were harvested and assayed for their ability to stimulate influenza-reactive CD8^+^ T cells. To bypass the requirement for CD4^+^ T cell help in the activation of CD8^+^ T cells via the exogenous pathway, IL-12 was added to the cultures. Spot-forming cells (SFCs) per 10^6^ T cells are reported. Data are representative of three experiments. Values are averages of triplicate wells with error bars indicating standard deviation.

To ensure that the influenza antigens being monitored required transporter activity for the generation of MHC I/pep complexes, we directly infected DCs prepared from TAP^−/−^ mice (Figure 1). As has previously been reported, no T cell activation was evident when infected TAP^−/−^ DCs were employed (Figure 1A and 1B). Furthermore, we established that the TAP^−/−^ DCs efficiently engulfed apoptotic bodies and that the kinetics of uptake were similar to those evident in wild-type (WT) DCs (Figure S2). DCs that had internalized dying cells were tested for their ability to cross-present antigen; in contrast to their ability to present antigen via the “classical” MHC I pathway, the TAP^−/−^ DCs were able to cross-present influenza antigen derived from MHC-mismatched apoptotic cells as efficiently as WT DCs (Figure 1D and 1E). To rule out the possibility that transporter activity was simply being transferred via fusion of membranes between the apoptotic cells and the DC during phagocytosis, we tested whether TAP^−/−^ DCs that had previously internalized uninfected TAP-expressing apoptotic cells could now present antigen after direct infection with influenza (flu-[TAP^−/−^ DC x/p 3T3]; Figure 1C). We detected background levels of T cell activation in these assays as compared to the robust stimulation observed using influenza-infected WT DCs (Figure 1C), arguing against such a mechanism and supporting the possibility that TAP^−/−^ DCs are capturing processed antigen from apoptotic cells.

**Figure 2:**
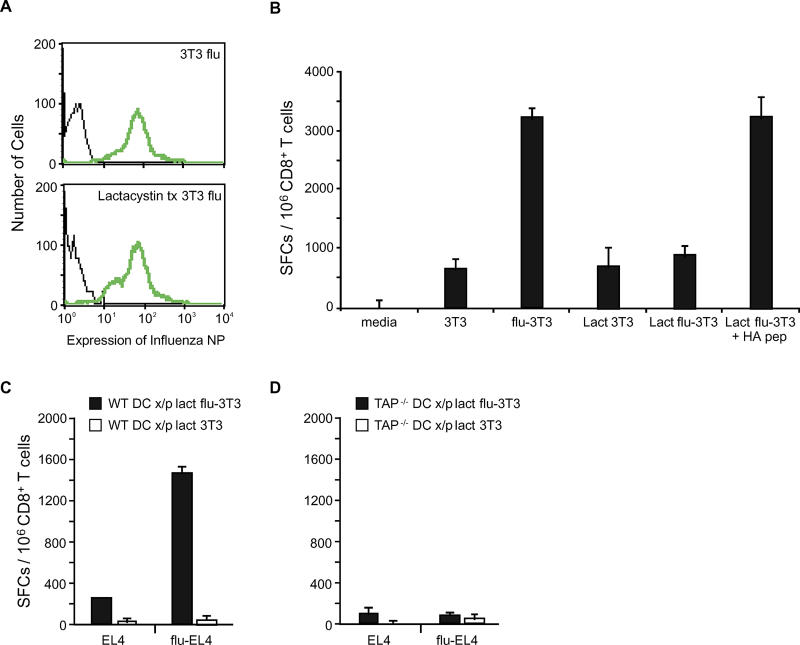
Processed Antigen from the Dying Cell Is Required for MHC I Presentation in TAP^−/−^ DCs To generate apoptotic cells lacking processed antigen, lactacystin pretreatment of influenza-infected H-2^d^ 3T3 cells was performed. Expression of influenza antigen was evaluated by intracellular FACS analysis using influenza NP mAbs followed by PE-conjugated goat anti-mouse mAb (A). Expression of MHC I/pep complexes in the lactacystin-treated 3T3 cells was evaluated by monitoring the activation of H-2^d^-restricted influenza hemagglutanin-reactive T cells. The K^d^-restricted immunodominant peptide (HA_210–219_) derived from hemagglutanin was pulsed onto 3T3 cells and served as a positive control (B). The influenza-infected H-2^d^ 3T3 cells were then induced to undergo apoptosis, and co-cultures were generated using C57BL/6 WT DCs (C) or TAP^−/−^ DCs (D). To evaluate T cell activation and expansion, DCs cross-presenting antigen were cultured with CD8^+^ T cells in the presence of IL-12 for 7–8 d. T cells were then harvested and tested for influenza reactivity in a 20-h IFN-γ ELISPOT. H-2^b^ EL4 cells with or without influenza infection served as the stimulators in the ELISPOT assay as above. Data are representative of two experiments. Values are averages of triplicate wells with error bars indicating standard deviation.

To further establish the role for antigen processing in the dying cell, we generated apoptotic cells that expressed influenza proteins but were unable to process the influenza viral antigens. Prior to infection with influenza and the induction of apoptosis, 3T3 cells were treated with the proteasome inhibitor lactacystin [20]. We found that the lactacystin-treated cells expressed levels of influenza antigen similar to untreated cells (Figure 2A). The inhibition of proteasome activity was confirmed functionally using treated cells as stimulators for HA-reactive T cells restricted to H-2^d^, the MHC haplotype of the dying 3T3 cells (Figure 2B). Parallel cultures were triggered to undergo apoptosis and were co-cultured with WT or TAP^−/−^ DCs as described in the Materials and Methods. Cross-presentation of antigen by the DCs was evaluated based on the activation of influenza-reactive CD8^+^ T cells. We found that the lactacystin-treated apoptotic cells were competent to serve as a source of antigen for WT DCs (Figure 2C); however, the lack of processed antigen in lactacystin-treated 3T3 cells prevented cross-presentation by TAP^−/−^ DCs (Figure 2D). This result further establishes that transporter activity is not passed from the dying cell to the TAP^−/−^ DC as a result of an ill-defined fusion event. Instead, it is an active process whereby processed antigen within the dying cell is being utilized by the DC for the generation of MHC I/pep complexes.

**Figure 3:**
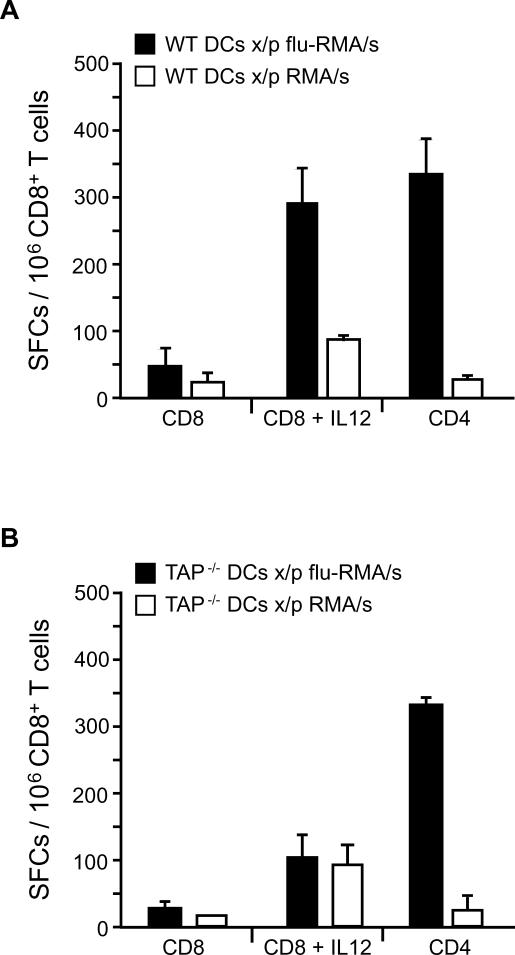
Transporter Activity Is Required in Either the Apoptotic Cell or the DC for Efficient Cross-Presentation of Antigen RMA/S cells were infected with influenza and irradiated with UVB to allow for antigen loading and the induction of apoptotic death. Co-cultures were generated as described in the Materials and Methods using WT DCs (A) or TAP^−/−^ DCs (B). DCs were harvested and tested for their ability to cross-present antigen and activate influenza-reactive CD8^+^ T cells as measured in a 40-h ELISPOT assay. To evaluate loading of MHC II, WT DCs and TAP^−/−^DCs that had captured apoptotic antigen were tested for their ability to activate influenza-reactive CD4+ T cells. Data are representative of four experiments. Values are averages of triplicate wells with error bars indicating standard deviation.

To determine the importance of antigen access to the dying cell's endoplasmic reticulum (ER), we used RMA/s cells, which are deficient in TAP-2, as a source of influenza antigen. These cells express influenza proteins upon infection (Figure S3A), but do not facilitate peptide transport into the ER, as established by their inability to re-stimulate an influenza-reactive CD8^+^ T cell line (Figure S3B). When WT DCs were employed as the APC, RMA/s served as a source of antigen for the generation of MHC I/pep complexes (Figure 3A); in contrast, when TAP^−/−^ DCs were used, no activation of CD8^+^ T cells was observed (Figure 3B). While the absence of TAP in both the dying cell and the DC prevented loading of MHC I, antigen presentation on MHC II was unaffected, as equivalent stimulation of influenza-reactive CD4^+^ T cells was observed when using WT or TAP^−/−^ DCs (Figure 3A and 3B).

Together, these results suggest the presence of two independent pathways by which antigen may be cross-presented. The first is a pathway that requires transporter activity in the DC—presumably relying on the transport of exogenous antigen from the phagosome to the cytosol—and accounts for the requirement for TAP-sufficient DCs to generate MHC I/pep complexes in conditions where the peptide is derived from whole or partially degraded protein that is present within internalized apoptotic lactacystin-treated 3T3 cells (see Figure 2C) or TAP^−/−^ RMA/S cells (see Figure 3B). In the second, the DCs are able to capture processed antigen present within proteasome- and TAP-competent dying cells; notably, this latter antigen source may be cross-presented without a need for further transporter activity within the DC (see Figure 1E).

To evaluate the in vivo relevance of these findings, we took advantage of a recent observation made by Woodland and colleagues regarding the unique ability of DCs to generate MHC I peptides derived from the influenza A/PR/8 acid polymerase (PA) protein [21]. They demonstrated that while most cells are capable of processing influenza A/PR/8 nucleoprotein (NP), only DCs process and present the epitope PA_224–233_. We confirmed this result and demonstrated that both epitopes required transporter activity in the infected DC—in other words, influenza-infected TAP^−/−^ DCs activated neither NP_366–374_- nor PA_224–233_-specific T cells (data not shown).

**Figure 4:**
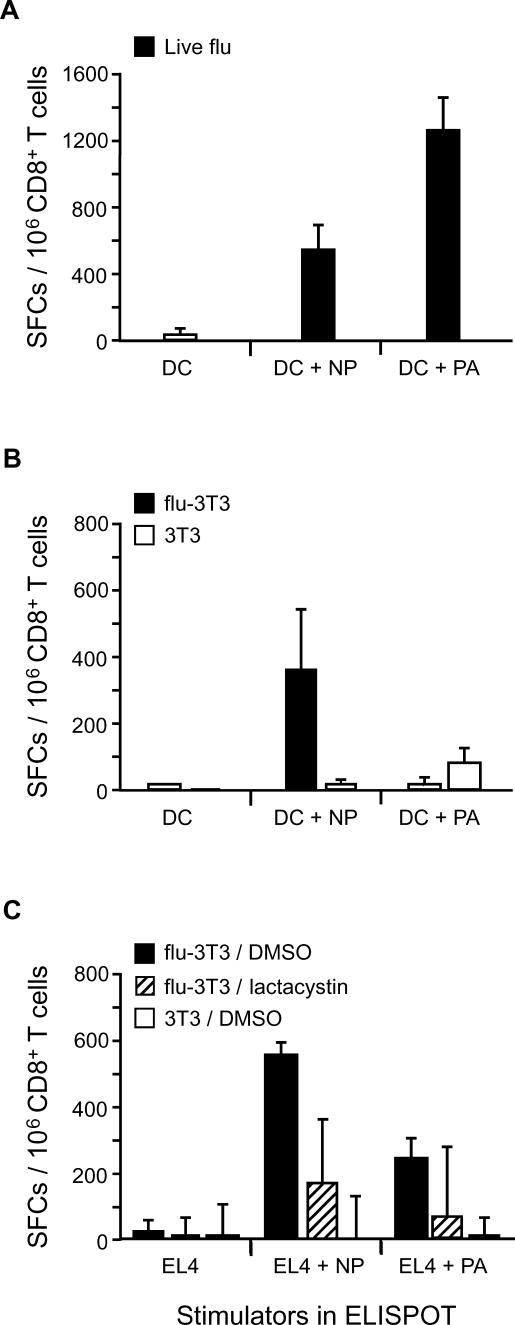
Immunization with Apoptotic Cells Results in the Selective Priming of T Cells Reactive to Processed Antigen (A and B) C57BL/6 mice were immunized intraperitoneally with 300 HAU of influenza (A), or 5 × 10^6^ infected apoptotic 3T3 cells (B). After 14 d, splenocytes were harvested, and CD8^+^ T cells were purified. To assay for the specificity of these cells, an IFN-γ ELISPOT was performed using the following stimulators: DCs alone or DCs pulsed with either 1 μM NP_366–374_ or 1 μM PA_224–233_ peptide. (C) C57BL/6 mice were immunized intraperitoneally with 5 × 10^6^ untreated versus lactacystin-treated influenza-infected apoptotic 3T3 cells. As above, 14 d after priming, splenocytes were harvested, and CD8^+^ T cells were purified and assayed for their reactivity to NP_366–374_ versus PA_224–233_. In this experiment, peptide-pulsed EL4 cells were employed as the stimulators. Data are representative of two experiments. Values are averages of triplicate wells with error bars indicating standard deviation.

**Figure 5:**
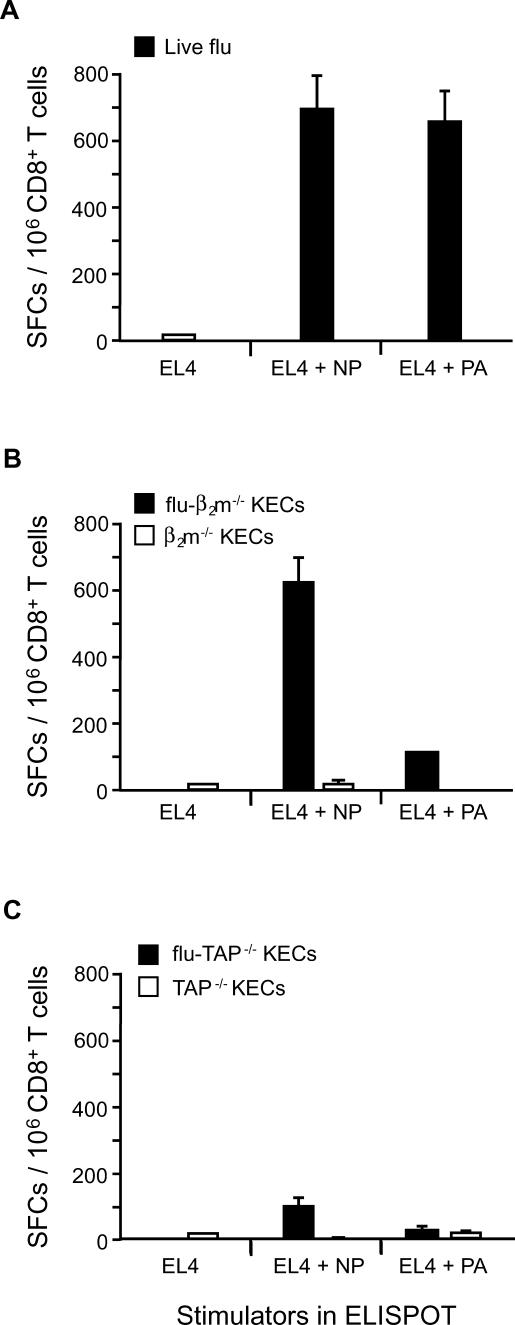
Processed Antigen within the Dying Cell Is Required for Efficient In Vivo Priming C57BL/6 mice were immunized intraperitoneally with 300 HAU of influenza (A), or 2 × 10^6^ infected apoptotic kidney epithelial cells derived from β_2_m-deficient (B) or TAP-deficient mice (C). After 14 d, splenocytes were harvested, and CD8^+^ T cells were purified. To assay for the specificity of these cells, an IFN-γ ELISPOT was performed using the following stimulators: EL4 cells alone or EL4 cells pulsed with either 1 μM NP_366–374_ or 1 μM PA_224–233_ peptide. Values are averages of triplicate wells with error bars indicating standard deviation.

We next analyzed the T cell repertoire generated after priming C57BL/6 (H-2^b^) mice with live influenza versus influenza-infected apoptotic 3T3 (H-2^d^) cells. Given that the apoptotic cells express both NP and PA protein, but process only NP_366–374_, the in vivo activation of NP_366–374_- and PA_224–233_-specific T cells would suggest that the DC was responsible for processing the antigen, whereas a response to NP_366–374_ in the absence of a response to PA_224–233_ would imply that processed antigen was the preferred source of antigen for cross-presentation. As reported by Crowe et al. [21], priming the mice with 300 hemaglutanin units (HAU) of live influenza resulted in the activation of both NP_366–374_- and PA_224–233_-reactive T cells (Figure 4A). However, when apoptotic influenza-infected 3T3 cells were injected into naïve mice, we observed a robust NP_366–374_-specific response and only weak reactivity to PA_224–233_ (Figure 4B). To examine whether the response was indeed due to the processing of antigen within the dying cell, we primed naïve mice using lactacystin-treated influenza-infected 3T3 cells (prepared as above), and observed a marked reduction in the efficiency of NP_366–374_-reactive T cell priming (Figure 4C). Similarly, we demonstrated that infected kidney epithelial cells derived from β_2_m-deficient but not TAP^−/−^ mice were capable of priming NP_366–374_-specific T cells (Figure 5). These data again highlight the importance of processed antigen within the ER of the dying cell. In sum, we demonstrated the in vivo relevance of DCs capturing processed antigen derived from dying cells for the cross-priming of CD8^+^ T cells, and suggest that in some experimental conditions, this pathway may be more efficient than the cross-priming of whole protein.

## Discussion

Several models for antigen cross-presentation have evaluated a role for transporter activity, and most report that COOH terminal processing by the proteasome and utilization of TAP by the APC is essential [22,23]. This is understandable when exogenous antigen is derived from internalized immune complexes [18], antigen-coated latex beads [16,17], or whole protein [19]. In these instances, there is no ability for antigen processing to occur in a manner that would permit loading of DC MHC I in the absence of transporter activity. With respect to in vitro systems that have reported a TAP-independent pathway, the antigens successfully presented seem limited to peptides immediately COOH-terminal to an ER targeting sequence, or those within secreted or transmembrane proteins that are processed by still undefined ER proteases [24,25]. It has also been demonstrated that at high levels of antigen challenge, it is possible for peptide epitopes to be generated by Cathepsin S within the phagolysosome [26].

**Figure 6:**
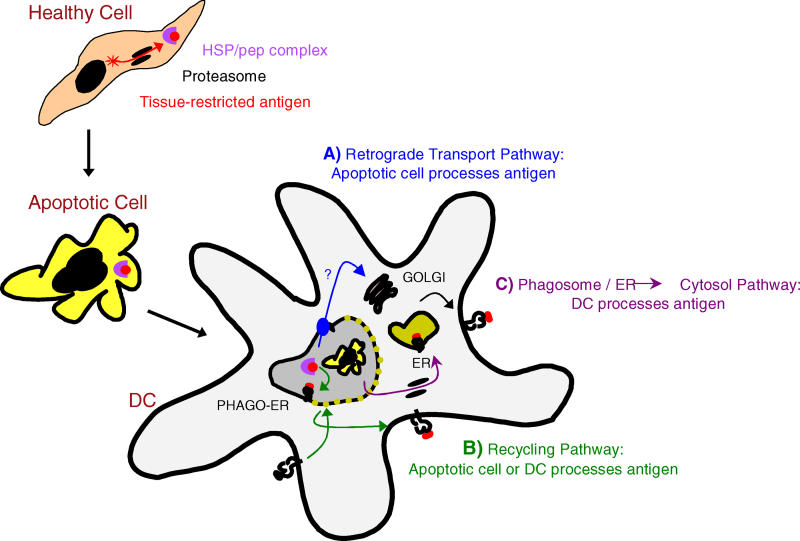
An Active Role for Apoptotic Cells in the Transfer of Antigen to DCs We propose that apoptotic cells play an active role through the transfer of processed antigen to DCs for the generation of MHC I/pep complexes. This pathway may be dominant in the presentation of infectious antigen as the virus may co-opt cellular translational machinery, resulting in high levels of viral protein, and the upregulation of stress proteins, as well as inducing apoptotic cell death. Defective ribosomal initiation products chaperoned by HSPs offer a potential source of antigen. Within the DC, HSPs derived from the internalized apoptotic cell may traffic via a retrograde transport pathway, shuttled to the *trans*-Golgi and then the ER via binding to KDEL receptors (A). Alternatively, the evidence for phagosome–ER (PHAGO-ER) fusion and/or the recycling of MHC I from the plasma membrane offers the possibility that processed antigen may interact directly with the DC's MHC I (B). As ER chaperones within the phagocytosed cell would be bound to the pool of peptides derived from newly synthesized proteins, these pathways offer the DC an accurate representation of what occurred immediately prior to death (A and B). At high concentrations of protein, we also find evidence for the DC processing the cross-presented antigen. This likely occurs via a phago–ER-to-cytosol pathway as has been previously described (C).

In this study, we restricted our analysis to physiologically relevant levels of antigen (all of which require proteasome processing and TAP activity), and asked whether dying cells serve as a source of whole protein or whether they may also participate in antigen presentation by generating processed antigen that may be transferred to DCs. As has been previously shown, our data demonstrated that WT DCs can process antigen from cells that contain whole or partially processed protein (see Figures 2C and 3A). To assess the ability of the dying cell to process the antigen, TAP^−/−^ DCs were used. Applying this strategy, we identified the existence of an antigen cross-presentation pathway that utilizes proteasome and transporter activity present in the dying cell (see Figures 1, 2, and 3). Importantly, when lactacystin-treated or TAP^−/−^ apoptotic cells were the source of antigen, the TAP^−/−^ DCs were no longer capable of cross-presenting antigen (see Figures 2D and 3B). Furthermore, we demonstrated activation of CD4^+^ T cells in all experimental conditions, illustrating that DCs indeed captured dying cells expressing influenza antigen, even in situations where MHC I presentation was inhibited. While this in vitro system allowed us to carefully control the nature of the antigen present in the dying cells (whole protein or processed antigen) and permitted us to ensure the transfer of cell-associated protein to the DCs, we also tested the ability to prime T cells in vivo. Taking advantage of the differential processing of influenza antigen by DCs versus other cell types, we demonstrated that in situations of direct infection, DCs processed the antigen. When dying cells were the source of antigen, we observed a preferential skewing of T cell cross-priming toward the protein that could be processed by the apoptotic cell. Lactacystin treatment of the influenza-infected cell or the use of TAP^−/−^ cells confirmed the requirement for proteasome processing and transporter activity within the dying cell (see Figures 4 and 5). Furthermore, the ability to cross-prime influenza-specific T cells with β_2_m-deficient but not TAP-deficient cells indicated that the antigen was originating from the ER of the dying cell (see Figure 5). When greater numbers of apoptotic cells were used (10–50×), it was possible to observe cross-priming of whole or partially degraded protein (data not shown). Our findings support the in vitro work shown here and that of Serna et al. [27]—cross-priming of influenza antigen favors the processed antigen within the dying cell (Figure 6). Indeed, apoptotic cells may play an active role in antigen presentation through the delivery of processed antigen, in turn allowing for efficient generation of MHC I/pep complexes by the DC.

The identification of this pathway raises the intriguing possibility that ER chaperones within the apoptotic cell are facilitating delivery of peptide epitopes to the DC (see Figure 6). Notably, the heat shock proteins (HSPs) GP96 and calreticulin have been shown to associate with newly processed cytosolic-derived epitopes, and when injected in vivo, they cross-prime cytotoxic T lymphocytes [28–30]. The recent reports of ER–phagosome fusion [31] suggest that HSP/peptide complexes may be capable of direct interaction with the DC's MHC I; alternatively, HSPs containing a KDEL motif (Lys-Asp-Glu-Leu) may employ a retrograde transport pathway to directly access the ER [24]. As ER chaperones within the phagocytosed cell would be bound to the pool of peptides derived from newly synthesized proteins [32], they offer the DC an accurate representation of what was being translated immediately prior to death. As an interesting alternative to HSPs, the processed antigen transferred may be the pool of peptides bound to chromatin [33]. As the nucleus lacks efficient peptidase activity, antigen may be protected within the nuclear remnants of an apoptotic body. If this were occurring, we predict that loading of the MHC I in the DC would occur in the phagolysosome.

Our findings are of particular interest when placed in the context of three recent studies that report that cell-associated whole protein is the primary source of antigen for cross-priming [13–15]. We fully appreciate that our study may reflect the choice of a viral model for cross-priming and acknowledge that the experimental details will influence the conclusions. In this light, it is important to consider the differences between the chosen model systems. In the work of Shen and Rock [13], lysates prepared from ovalbumin-transfected cell lines were used as a source of antigen, testing different subcellular fractions for their ability to prime animals. This study argues that intact cellular protein, rather than peptides or HSP/peptide complexes, is the main source of antigen for cross-presentation. Considering their use of nitrogen cavitation as the method for disrupting cells, which has been reported to dissociate antigenic peptides from HSP70 and GP96 [34,35], it would be expected that the HSPs within their lysates would indeed be inert. As a result, this model may have been biased toward the cross-presentation of whole antigen. Wolkers et al. [15] demonstrated that peptides present in the secretory domain of nascent proteins are not efficiently cross-presented, while the stable epitope within the mature protein is indeed transferred to APCs. The in vivo studies presented seem to be tracking the cross-presentation of secreted protein, not cell-associated protein. Use of H-2^d^ × H-2^b^ F_1_ mice to demonstrate cross-presentation from P815 cells supports the requirement for the P815 (H-2^d^) to remain alive. An alternate interpretation of their experiments is that soluble proteins produced (in large quantities) by growing tumors resulted in the observed in vivo T cell activation. As a result, there may have been little opportunity for processed cell-associated protein to gain access to a DC. Finally, the study from Norbury et al. [14] reported that proteasome substrates (rather than peptides) are critical for achieving antigen transfer for cross-presentation. These studies rely heavily on the use of lactacystin to inhibit proteasome activity. However, while they show the persistence of whole protein, they do not demonstrate that lactacystin prevents the generation of processed peptides in the experimental models used. In the studies described herein, 100 μM lactacystin with a maintenance dose of 1 μM, to inhibit newly synthesized proteasomes (during infection and antigen expression), is required to block the generation of peptides secondary to influenza infection. Given the possibility of newly synthesized proteasomes and/or proteasome-independent processing acting on their artificial constructs for the generation of peptide epitopes, it is critical that functional studies be used to exclude the production of small amounts of processed antigen.

In sum, while cell-associated whole protein is important in cross-priming, previous studies have not excluded proteasome products or HSP/peptide complexes as substrates for in vivo cross-priming. As shown here, the apoptotic cell may in fact play a critical role in processing antigen for cross-presentation, in essence preselecting immunologically important antigenic determinants. A comprehensive model accounting for antigen derived from whole protein as well as processed antigens from apoptotic cells is needed to more clearly define the pathways of antigen cross-priming in physiologic (resting) as well as pathologic (stress) situations.

## Materials and Methods

### 

#### Mice.

WT and TAP-1-deficient C57BL/6 mice were purchased from Jackson Laboratory (Bar Harbor, Maine, United States). In all experiments, 4- to 6-wk-old female mice were employed.

#### Antibodies, cell lines, and reagents.

All FACS antibodies used in this study were obtained from BD Biosciences Pharmingen (San Diego, California, United States); reagents for the ELISPOT assays were obtained from Mabtech (Stockholm, Sweden). PC3 cells, a human prostate cancer cell line, were obtained from American Type Culture Collection ( ATCC) (Manassas, Virginia, United States). BALB/3T3 cells clone A31 (3T3) were obtained from ATCC. RMA/S, a TAP-deficient T cell lymphoma cell line derived from the Rauscher murine leukemia virus–induced RBL-5 cell line, was employed [36]. β_2_m- and TAP-deficient kidney epithelial cells were derived from organ culture followed by a 2-wk in vitro expansion. All cell lines were grown in DMEM containing 10% fetal bovine serum, supplemented with nonessential amino acids, sodium pyruvate, glutamine, 2β-mercaptoethanol, and gentamicin (D-10). Human influenza A/PR/8 was provided as allantoic fluid from Charles River Laboratories (Wilmington, Massachusetts, United States) and used at a 1:3 dilution to infect PC3, 3T3, or RMA/S cells (1,000 HAU/10^6^ cells) or 1:10 dilution to infect DCs (300 HAU/10^6^ cells). Recombinant mouse TNF-α and IL-12 were obtained from R&D Systems (Minneapolis, Minnesota, United States).

#### Preparation of antigen-loaded DCs.

Bone-marrow-derived DCs were prepared as previously described [37]. In brief, bone marrow obtained from tibia and femurs was lysed of red blood cells and cultured at a density of 3 × 10^6^ cells/well in six-well plates with RPMI containing 10% fetal bovine serum, nonessential amino acids, sodium pyruvate, glutamine, 2β-mercaptoethanol, gentamicin (R-10), and in the presence of GM-CSF (provided by J558L cells transduced with GM-CSF, used 3% vol/vol). Fresh GM-CSF-supplemented medium was added to the wells on days 2, 4, and 6. On day 7, DCs were harvested and plated in fresh wells with or without apoptotic cells. In addition, a maturation stimulus, 80 ng/ml rmTNF-α, was added. To generate influenza-infected apoptotic cells, living cells were first infected with influenza for 1 h at 37 °C in serum-free medium. To allow for expression of viral proteins, 3–5 × 10^6^ infected cells per well of a six-well plate were cultured for 5 h at 37 °C. Cells were washed three times with 3 ml of PBS and were UVB irradiated (120 mJ/cm^2^) in 0.5 ml of PBS, and 0.5 ml of R-10 was added. Cells were allowed to undergo apoptosis for 8–10 h prior to adding 10^6^ immature DCs. Non-adherent cells were harvested 36 h later, and mature DCs were purified to greater than 95% purity using anti-CD11c microbeads and LS^+^ columns (Miltenyi Biotec, Bergisch Gladbach, Germany). DCs were monitored by FACS and found to express high levels of I-A^b^ and CD40. To generate influenza-infected DCs, day 9 mature DCs were infected with influenza for 1 h at 37 °C in serum-free medium. These cells were washed three times in serum containing medium, counted, and used directly.

#### In vitro cross-presentation studies.

Four- to six-week-old mice were infected intraperitoneally with 200–300 HAU of influenza A/PR/8–1976 (Charles River, North Franklin, Connecticut, United States). After 2–4 wk, CD4^+^ and CD8^+^ T cells were isolated using MACS purification (Miltenyi Biotec). These cells served as responders in antigen cross-presentation ELISPOT assays. Stimulators included WT or TAP^−/−^ DCs presenting antigen via the endogenous or exogenous pathway. To achieve a ratio of 30 T cells to one DC, 2 × 10^5^ T cells were added to 6.6 × 10^3^ DCs. Cultures were incubated in the plates for 20–36 h at 37 °C, after which cells were washed out of the ELISPOT plates using a mild detergent followed by incubation with 1 μg/ml biotin-conjugated α-interferon-γ (α-IFN-γ) monoclonal antibody (mAb) (BD Biosciences Pharmingen, clone XMG1.2). Wells were then developed using the Vectastain Elite Kit as per manufacturer's instructions (Vector Laboratories, Burlingame, California, United States). Colored spots represent IFN-γ-releasing cells and are reported as spot-forming cells per 10^6^ cells. The ELISPOT plate evaluation was performed in a blinded fashion by an independent evaluation service (ZellNet Consulting, Fort Lee, New Jersey, United States) using an automated ELISPOT reader (Carl Zeiss, Thornwood, New York, United States) with KS Elispot 4.3 software.

#### In vivo cross-priming studies.

Mice were primed intraperitoneally using influenza A/PR/8-infected apoptotic cells. Ten to 14 d post-immunization, spleens were harvested and CD8^+^ T cells were isolated using MACS purification (Miltenyi Biotec). A total of 2 × 10^5^ T cells were added to 2 × 10^4^ peptide-pulsed DCs or EL4 cells (haplotype-matched cell line) in the ELISPOT plates, pre-coated with 5 μg/ml of a primary α-IFN-γ mAb (Mabtech, clone AN18). Cultures were incubated for 20–36 h at 37 °C, and developed as above.

## Supporting Information

Figure S1 DC Cross-Presenting Apoptotic Cells Prime CD8^+^ T Cells In Vivo and Serve As Targets for Effector CD8^+^ T Cells In Vitro (A) C57BL/6 mice were immunized intra-footpad with 10^5^ DCs cross-presenting apoptotic, influenza-infected PC3 cells, or apoptotic influenza-infected PC3 cells alone. Six days following this single immunization, draining lymph nodes were harvested, CD8^+^ T cells were immediately purified and tested for their ability to respond to syngeneic stimulator cells with or without antigen in a 20-hr IFN-γ ELISPOT assay. SFCs per 10^6^ CD8+ T cells are reported.
(B) As shown in the schematic representation, surface expression of influenza-peptide-loaded MHC I by DCs was monitored using a modified cytotoxicity assay. After charging the DCs with antigen via the endogenous or exogenous pathways, they were loaded with ^51^Cr and used as targets for previously activated influenza-specific cytotoxic T lymphocytes. This assay is designed to evaluate the surface expression of MHC I/pep complexes. DCs were loaded via the exogenous pathway with influenza-infected allogeneic 3T3 cells (filled black squares) or uninfected 3T3 cells (open black squares). Alternatively, DCs were directly infected, thus presenting antigen via the endogenous pathway (filled red squares) or left uninfected (open red squares). After antigen expression or capture of the apoptotic material, respectively, DCs were loaded with ^51^Cr and tested as targets. After 5 h, supernatants were collected and percent cytotoxicity was calculated. Percent cytotoxicity = (experimental-well ^51^Cr release − spontaneous release)/(total release − spontaneous release) × 100.
(95 KB EPS).

Figure S2 TAP^−/−^ DCs Efficiently Phagocytose Apoptotic Cells Immature DCs from C57BL/6 WT or TAP-1-deficient (TAP^−/−^) mice were prepared as above and labeled with the PKH-67-GL (green) fluorescent cell linker (A and B). These cells were next added to PKH-26-GL-labeled (red) apoptotic cells (ACs) for 7 h at a ratio of one DC to five apoptotic cells in the presence or absence of EDTA. FACS Calibur analysis allowed for the detection of double-positive cells (A), indicating that the green DCs captured red apoptotic material. Phagocytosis was calculated as the percent double-positive cells per total population of DCs (A). Samples of DCs alone and apoptotic cells alone were used for setting the parameters of the flow cytometer. The kinetics of phagocytosis were monitored throughout the experiment, and the percent double-positive cells is reported (B).
(54 KB PPT).

Figure S3 RMA/s Cells Express but Do Not Present Influenza Antigen on MHC I (A) RMA or RMA/S cells were infected with influenza and incubated at 37 °C for 5 h to allow for viral antigen expression. Cells were fixed, permeabilized, and stained using NP mAbs followed by PE-conjugated goat anti-mouse mAb. Analysis was performed on a FACS Calibur, and histograms are shown.
(B) Infected RMA or RMA/s cells were used as stimulators in an ELISPOT assay, testing for their ability to stimulate influenza-reactive CD8^+^ T cells. The influenza A/PR/8 NP_366–374_ peptide restricted for D^b^ was pulsed onto RMA or RMA/s cells and served as a positive control. Values are averages of triplicate wells with error bars indicating standard deviation.
(205 KB EPS).

Protocol S1 DCs Capture Apoptotic Cells and Cross-Present Antigen to CD8^+^ T Cells (31 KB DOC).

### Accession Numbers

The NCBI Entrez Protein (http://www.ncbi.nlm.nih.gov/entrez/query.fcgi?db=Protein) accession numbers for the gene products discussed in this paper are influenza PA (AAA43619), influenza NP (B36754), and K^d^-restricted immunodominant peptide (HA_210–219_) derived from hemagglutanin (NP040980).

## Abbreviations

APC: antigen-presenting cell
DC: dendritic cell
ER: endoplasmic reticulum
HAU: hemaglutanin units
HSP: heat shock protein
IFN-γ: interferon-γ
IL-12: interleukin-12
MHC: major histocompatibility
mAb: monoclonal antibody
MHC I/pep: major histocompatibility class I/peptide
NP: A/PR/8 nucleoprotein
PA: A/PR/8 acid polymerase
WT: wild-type

## References

[pbio-0030185-b01] Savill J (1997). Recognition and phagocytosis of cells undergoing apoptosis. Br Med Bull.

[pbio-0030185-b02] Guermonprez P, Valladeau J, Zitvogel L, Thery C, Amigorena S (2002). Antigen presentation and T cell stimulation by dendritic cells. Annu Rev Immunol.

[pbio-0030185-b03] Heath WR, Carbone FR (2001). Cross-presentation in viral immunity and self-tolerance. Nat Rev Immunol.

[pbio-0030185-b04] Albert ML, Darnell JC, Bender A, Francisco LM, Bhardwaj N (1998). Tumor-specific killer cells in paraneoplastic cerebellar degeneration. Nat Med.

[pbio-0030185-b05] Albert ML, Pearce SF, Francisco LM, Sauter B, Roy P (1998). Immature dendritic cells phagocytose apoptotic cells via alphavbeta5 and CD36, and cross-present antigens to cytotoxic T lymphocytes. J Exp Med.

[pbio-0030185-b06] Albert ML, Sauter B, Bhardwaj N (1998). Dendritic cells acquire antigen from apoptotic cells and induce class I-restricted CTLs. Nature.

[pbio-0030185-b07] Bevan MJ (1976). Cross-priming for a secondary cytotoxic response to minor H antigens with H-2 congenic cells which do not cross-react in the cytotoxic assay. J Exp Med.

[pbio-0030185-b08] Albert ML, Jegathesan M, Darnell RB (2001). Dendritic cell maturation is required for the cross-tolerization of CD8+ T cells. Nat Immunol.

[pbio-0030185-b09] Heath WR, Kurts C, Miller JF, Carbone FR (1998). Cross-tolerance: A pathway for inducing tolerance to peripheral tissue antigens. J Exp Med.

[pbio-0030185-b10] Kurts C, Carbone FR, Barnden M, Blanas E, Allison J (1997). CD4+ T cell help impairs CD8+ T cell deletion induced by cross-presentation of self-antigens and favors autoimmunity. J Exp Med.

[pbio-0030185-b11] Albert ML, Kim JI, Birge RB (2000). α_v_β_5_ integrin recruits the CrkII-Dock180-rac1 complex for phagocytosis of apoptotic cells. Nat Cell Biol.

[pbio-0030185-b12] Inaba K, Turley S, Yamaide F, Iyoda T, Mahnke K (1998). Efficient presentation of phagocytosed cellular fragments on the major histocompatibility complex class II products of dendritic cells. J Exp Med.

[pbio-0030185-b13] Shen L, Rock KL (2004). Cellular protein is the source of cross-priming antigen in vivo. Proc Natl Acad Sci U S A.

[pbio-0030185-b14] Norbury CC, Basta S, Donohue KB, Tscharke DC, Princiotta MF (2004). CD8+ T cell cross-priming via transfer of proteasome substrates. Science.

[pbio-0030185-b15] Wolkers MC, Brouwenstijn N, Bakker AH, Toebes M, Schumacher TN (2004). Antigen bias in T cell cross-priming. Science.

[pbio-0030185-b16] Guermonprez P, Saveanu L, Kleijmeer M, Davoust J, Van Endert P (2003). ER-phagosome fusion defines an MHC class I cross-presentation compartment in dendritic cells. Nature.

[pbio-0030185-b17] Houde M, Bertholet S, Gagnon E, Brunet S, Goyette G (2003). Phagosomes are competent organelles for antigen cross-presentation. Nature.

[pbio-0030185-b18] Rodriguez A, Regnault A, Kleijmeer M, Ricciardi-Castagnoli P, Amigorena S (1999). Selective transport of internalized antigens to the cytosol for MHC class I presentation in dendritic cells. Nat Cell Biol.

[pbio-0030185-b19] Pooley JL, Heath WR, Shortman K (2001). Cutting edge: Intravenous soluble antigen is presented to CD4 T cells by CD8− dendritic cells, but cross-presented to CD8 T cells by CD8+ dendritic cells. J Immunol.

[pbio-0030185-b20] Fenteany G, Standaert RF, Lane WS, Choi S, Corey EJ (1995). Inhibition of proteasome activities and subunit-specific amino-terminal threonine modification by lactacystin. Science.

[pbio-0030185-b21] Crowe SR, Turner SJ, Miller SC, Roberts AD, Rappolo RA (2003). Differential antigen presentation regulates the changing patterns of CD8+ T cell immunodominance in primary and secondary influenza virus infections. J Exp Med.

[pbio-0030185-b22] Pamer E, Cresswell P (1998). Mechanisms of MHC class I-restricted antigen processing. Annu Rev Immunol.

[pbio-0030185-b23] Yewdell JW, Bennink JR (1998). TAP-independent delivery of antigenic peptides to the endoplasmic reticulum: Therapeutic potential and insights into TAP-dependent antigen processing. J Immunother.

[pbio-0030185-b24] Castellino F, Boucher PE, Eichelberg K, Mayhew M, Rothman JE (2000). Receptor-mediated uptake of antigen/heat shock protein complexes results in major histocompatibility complex class I antigen presentation via two distinct processing pathways. J Exp Med.

[pbio-0030185-b25] Snyder HL, Bacik I, Bennink JR, Kearns G, Behrens TW (1997). Two novel routes of transporter associated with antigen processing (TAP)-independent major histocompatibility complex class I antigen processing. J Exp Med.

[pbio-0030185-b26] Shen L, Sigal LJ, Boes M, Rock KL (2004). Important role of cathepsin S in generating peptides for TAP-independent MHC class I crosspresentation in vivo. Immunity.

[pbio-0030185-b27] Serna A, Ramirez MC, Soukhanova A, Sigal LJ (2003). Cutting edge: Efficient MHC class I cross-presentation during early vaccinia infection requires the transfer of proteasomal intermediates between antigen donor and presenting cells. J Immunol.

[pbio-0030185-b28] Basu S, Srivastava PK (1999). Calreticulin, a peptide-binding chaperone of the endoplasmic reticulum, elicits tumor- and peptide-specific immunity. J Exp Med.

[pbio-0030185-b29] Blachere NE, Li Z, Chandawarkar RY, Suto R, Jaikaria NS (1997). Heat shock protein-peptide complexes, reconstituted in vitro, elicit peptide-specific cytotoxic T lymphocyte response and tumor immunity. J Exp Med.

[pbio-0030185-b30] Suto R, Srivastava PK (1995). A mechanism for the specific immunogenicity of heat shock protein-chaperoned peptides. Science.

[pbio-0030185-b31] Gagnon E, Duclos S, Rondeau C, Chevet E, Cameron PH (2002). Endoplasmic reticulum-mediated phagocytosis is a mechanism of entry into macrophages. Cell.

[pbio-0030185-b32] Lammert E, Arnold D, Nijenhuis M, Momburg F, Hammerling GJ (1997). The endoplasmic reticulum-resident stress protein gp96 binds peptides translocated by TAP. Eur J Immunol.

[pbio-0030185-b33] Reits E, Griekspoor A, Neijssen J, Groothuis T, Jalink K (2003). Peptide diffusion, protection, and degradation in nuclear and cytoplasmic compartments before antigen presentation by MHC class I. Immunity.

[pbio-0030185-b34] Srivastava PK (1997). Purification of heat shock protein-peptide complexes for use in vaccination against cancers and intracellular pathogens. Methods.

[pbio-0030185-b35] Peng P, Menoret A, Srivastava PK (1997). Purification of immunogenic heat shock protein 70-peptide complexes by ADP-affinity chromatography. J Immunol Methods.

[pbio-0030185-b36] Karre K, Ljunggren HG, Piontek G, Kiessling R (1986). Selective rejection of H-2-deficient lymphoma variants suggests alternative immune defence strategy. Nature.

[pbio-0030185-b37] Inaba K, Inaba M, Romani N, Aya H, Deguchi M (1992). Generation of large numbers of dendritic cells from mouse bone marrow cultures supplemented with granulocyte/macrophage colony-stimulating factor. J Exp Med.

