# Supplementary material for: Apoptotic Cells Deliver Processed Antigen to Dendritic Cells for Cross-Presentation
Source: PLoS Biol. 2005 Apr 26;3(6):e185. doi: 10.1371/journal.pbio.0030185 (PMC1084338; doi:10.1371/journal.pbio.0030185)
Supplement: Supplementary file 1 [file pbio.0030185.sd001.doc]

**Protocol S1. DCs capture apoptotic cells and cross-present antigen to CD8^+^ T cells.**

To establish a cross-priming system in mice and assess the *in vivo* potency of apoptotic cells, we immunized mice and assayed the primary expansion of MHC I-restricted CD8^+^ T cells. Human PC3 cells infected with influenza were induced to undergo apoptotic cell death using UV-B irradiation, and served as a source of exogenous antigen. PC3 cells were employed as they do not themselves result in cross-priming of CD8^+^ T cells (see below). Bone-marrow derived DCs were co-cultured with the infected apoptotic PC3 cells in the presence of TNF-α, which triggered DC maturation as confirmed by increased expression of CD86 and MHC II (data not shown). Mice were immunized with DCs cross-presenting the infected apoptotic PC3 cells, and one week later, CD8^+^ T cells were purified from draining lymph nodes and the frequency of influenza-reactive cells was determined using an interferon-γ (IFN-γ) ELISPOT assay. As shown, a single injection of 10^5^ DCs cross-presenting apoptotic cells efficiently primed CD8^+^ T cells (Figure S1A). Influenza-infected apoptotic PC3 cells do not harbor virus competent to permit direct infection *in vivo* (Figure S1A), and the T cell response was antigen-dependent, as immunization with uninfected apoptotic cells gave no response (data not shown). These results demonstrate that a single injection is sufficient to cross-prime naïve antigen-reactive T cells.

In order to dissect the mechanism(s) by which antigens derived from apoptotic cells are processed and presented by DCs, we next established an *in vitro* system that would permit us to control which cell (the apoptotic cell *versus* the dendritic cell) possessed the capacity to process antigen. Mice were infected with influenza in order to generate a population of memory T cells that could serve as a read-out for DC cross-presentation assays. Bulk CD8^+^ and CD4^+^ T cells were isolated from the spleen of mice two weeks post-immunization. This methodology allowed us to study the activation of a physiologically relevant population of antigen-reactive T cells within the context of the normal T cell repertoire (see methods). Based on >75 experiments using pooled groups of mice infected with influenza virus, the precursor frequency of memory CD8^+^ T cells post-infection is 200-1400 per million bulk CD8^+^ T cells. Bone marrow-derived DCs were co-cultured with apoptotic, influenza-infected allogeneic 3T3 cells (H-2^d^) in the presence of a maturation stimulus, purified based on expression of CD11c, and placed into ELISPOT wells with highly purified CD8^+^ T cells. As observed in our human studies(Albert et al. 2001), direct infection of DCs results in the activation of CD8^+^ and CD4^+^ T cells (Figure S1B), while DCs cross-presenting exogenous antigen failed to stimulate purified CD8^+^ T cells (Figure S1C). Antigen capture was not limiting as activation of MHC II-restricted CD4^+^ T cells could be observed (Figure S1C). This confirmed that activation of CD8^+^ T cells via the exogenous MHC I pathway requires CD4^+^ T cell help, which is believed to act *via* the DC through engagement of CD40 and other receptors of the TNF superfamily (Albert et al. 2001; Bennett et al. 1998; Ridge et al. 1998; Schoenberger et al. 1998). In prior studies (Albert et al. 2001), we have shown that activating antibodies specific for CD40 or the addition of interleukin-12 (IL-12) to the DC / CD8^+^ T cell cultures successfully bypasses the requirement for help (Figure S1C and unpublished data). Moreover, there is an absolute requirement for internalization and cross-presentation by the DC, as ‘mixed’ cultures of DCs and apoptotic cells did not permit activation of CD8^+^ T cells (Figure S1D). In these studies, the role for IL-12 (or help) in facilitating activation via the cross-presentation pathway, may be due to (i) enhancing the sensitivity of primed CD8^+^ T cells; (ii) augmenting APC function of the DC; and/or (iii) licensing the DC to activate CD8^+^ T cells specific for cross-presented exogenous antigen.

To establish that the DCs were expressing surface MHC I/pep complexes, we generated an influenza antigen-specific CTL line and tested its ability to target DCs loaded with antigen via the endogenous *versus* exogenous pathways. We demonstrate that nearly 100% of the directly infected DCs could serve as targets, indicating that they had generated influenza MHC I/pep complexes on their surface. Similarly, the DCs cross-presenting antigen derived from internalized apoptotic cells were efficiently targeted. The maximal killing achieved in the experiment reported was 80 percent (Figure S3), a finding consistent with the fact that in a typical experiment 60-85 percent of DCs capture at least one apoptotic cell, as evaluated using a FACS-based phagocytosis. Establishing this *in vitro* model permitted characterization of the pathways by which antigens derived from apoptotic cells are processed and cross-presented by DCs for the surface expression of MHC I/pep complexes.
